# Metabolic Regulation Effect and Potential Metabolic Biomarkers of Pre-Treated Delphinidin on Oxidative Damage Induced by Paraquat in A549 Cells

**DOI:** 10.3390/foods11223575

**Published:** 2022-11-10

**Authors:** Yongli Ye, Jian Ji, Yaoguang Huang, Yinzhi Zhang, Xiulan Sun

**Affiliations:** State Key Laboratory of Food Science and Technology, School of Food Science and Technology, Collaborative Innovation Center of Food Safety and Quality Control, Jiangnan University, Wuxi 214122, China

**Keywords:** delphinidin, metabolomics, paraquat, A549 cell, antioxidant activity

## Abstract

Delphinidin (Del) is an anthocyanin component with high in vitro antioxidant capacity. In this study, based on the screening of a cell model, gas chromatography-time of flight mass spectrometry (GC-TOF/MS) was used to evaluate the effect of Del pre-protection on the metabolite levels of intracellular oxidative stress induced by paraquat (PQ). According to the cytotoxicity and reactive oxygen species (ROS) responses of four lung cell lines to PQ induction, A549 cell was selected and treated with 100 μM PQ for 12 h to develop a cellular oxidative stress model. Compared with the PQ-induced group, the principal components of the Del pretreatment group had significant differences, but not significant with the control group, indicating that the antioxidant activity of Del can be correlated to the maintenance of metabolite levels. Del preconditioning protects lipid-related metabolic pathways from the disturbance induced by PQ. In addition, the levels of amino acid- and energy-related metabolites were significantly recovered. Del may also exert an antioxidant effect by regulating glucose metabolism. The optimal combinations of biomarkers in the PQ-treatment group and Del-pretreatment group were alanine-valine-urea and alanine-galactose-glucose. Cell metabolome data provided characteristic fingerprints associated with the antioxidant activity of Del.

## 1. Introduction

Delphinidin (Del) is one of the main components of six anthocyanins in dark pigmented fruits, vegetables, and herbal plants, accounting for about 12%. Many studies have shown that Del has a variety of significant biological functions, including antioxidant [1,2,3], anti-inflammatory, antinociception [4], antiangiogenesis, and anticancer [5,6] owing to the presence of many active hydroxyl groups in its structure. The antioxidant protective effect of Del on endothelial cells can be attributed to the upregulation of intracellular total glutathione level by Del itself or by its degradation product gallic acid [7]. Del exerts its antioxidant activity by directly or indirectly affecting the levels of related intracellular regulatory factors involved in maintaining redox balance. At present, studies on Del at the cellular and animal levels have mainly focused on the bioavailability, metabolite identification, and specific signaling pathway regulation mechanism; the effect of anthocyanins on the biological metabolism level of their various functions and activities has rarely been studied [8,9]. Notably, only focusing on the bioavailability of anthocyanins or the signal pathways related to a certain redox system is not sufficient, which might lead to missing useful information about the regulation of various intracellular biochemical reactions.

In recent years, metabolomics based on cell culture in vitro has become an important and valuable tool for drug screening, toxicological, and functional evaluation [10,11,12]. Metabolomic analysis can show the overall physiological and biochemical function or status of biological systems, providing critical biological information about the relationship between the object and level changes of metabolites in a specific cell line [13]. The identification of potential biomarkers to evaluate the subtle physiological stress reflected by the organism can provide meaningful information for evaluating the function of compounds in the whole organism [14]. For instance, Dai et al. [15] studied the mechanism of potential recovery ability of soybean peptide TTYY to oxidative damage in rats by metabolomics, and they identified the potential biomarkers in urine. Moreover, the biomarkers of anti-inflammatory activity screened and identified by untargeted metabolomics and artificial neural network models can be used to predict new natural anti-inflammatory compounds [16]. Increasing attention has been paid to the relationship between biological or cellular metabolic dynamics and redox balance caused by active compounds or oxygen promoters [17,18,19]. However, there is limited information about the antioxidant mechanism of Del from the perspective of cell metabolomics.

In this study, the level changes in intracellular metabolites were analyzed, and the potential biomarkers were quantified after the Del preintervention and followed by oxidative stress. To obtain a cell line suitable for the oxidative stress model, the sensitivities of several lung cancer cell lines to induction with paraquat (PQ) were compared. A comprehensive nontargeted metabolomics strategy based on GC-TOF/MS was used to identify and quantify the level changes in the metabolites of cells with PQ-induced oxidative stress after Del pretreatment. Significantly different metabolites and the potential metabolic pathways were deduced and identified through multivariate statistical and metabolic pathway analyses, and potential biomarkers with high discriminant ability were identified by predictive models and algorithms and validated. The antioxidant mechanism of Del on the selected cell line was evaluated from the perspective of metabolomics, and the potential biomarkers were identified, thus providing a new perspective to better understand the antioxidant mechanism of active compounds.

## 2. Materials and Methods

### 2.1. Materials and Reagents

Human lung adenocarcinoma (NCI-H1395) cells, human embryonic lung fibroblast (HFL1) cells, human lung squamous carcinoma (SK-MES-1) cells, human lung adenocarcinoma epithelial cells (A549), and human large cell lung cancer (NCI-H661) cells were obtained from the Chinese Academy of Sciences (Shanghai, China). The cell culture media and fetal bovine serum were obtained from Gibco Laboratories (Gaithersburg, MD, USA). Del (purity ≥ 98%) was purchased from Shanghai Yuanye Bio-Technology Co., Ltd. (Shanghai, China).

PQ, ethoxyamine hydrochloride, N-methyl-N-trimethylsilyl trifluoroacetamide (MSTFA) (containing 1% trimethylchlorosilane, v/v), fatty acid standard (C8–C24), and 2,7-dichlorodihydrofluorescein diacetate (H2DCF-DA) were supplied by Sigma-Aldrich Chemical (St. Louis, MO, USA). The kits for BCA protein assay and CCK-8 assay were purchased from Beyotime Biotechnology Co., Ltd. (Shanghai, China). HPLC-grade methanol, isopropyl alcohol, acetonitrile, other analytical-grade chemicals, and Milli-Q-quality water (Millipore Corp., Bedford, MA, USA) were also used.

### 2.2. Cell Culture and Sample Preparation

Five cell lines were cultured to the continuous logarithmic growth phase in their optimal complete medium at 37 °C in 5% CO_2_. First, 100 μM PQ was added to induce cells for 12 h and 24 h after the cells grew to 80% confluence, respectively. A sensitive cell line was selected according to the results of cytotoxicity and reactive oxygen species (ROS) level. A PQ concentration range of 0–400 μM was designed to determine the induced dose of oxidative stress injury model. Subsequently, the selected cell line was grown to 80% confluence and was pretreated with the Del for 12 h, and the PQ was added for another 12 h with the optimal concentration. According to the preliminary experimental results, Del treatment within the range of 80 μM showed no significant cytotoxicity, and it showed high in vitro antioxidant capacity. Combined with the low bioavailability of anthocyanins, 40 μM concentration was finally selected as the preintervention concentration. Cells for cell viability and ROS analysis were cultured in 96-well plates, and for metabolomics analysis, they were cultured in 6-well plates (at least 1 × 10^7^ cells/well). Each group was designed with six replicates.

### 2.3. Cell Viability and ROS Assay

The cell survival rate and ROS generation were measured using a CCK-8 kit and the H2DCF-DA fluorescence probe (20 μM) following the manufacturer’s protocols. The experiments were independently performed three times; each group had three parallel runs at a time. Protein was quantified using a BCA protein assay kit and compared with the control group. The cell viability was obtained as follows: Cell activity/% = (OD_450 nm treatment group_ − OD_450 nm blank_)/(OD_450 nm control group_ − OD_450 nm blank_) × 100. ROS were detected in A549 cells with or without the Del treatment as described in our previous study [20].

### 2.4. Metabolite Analysis

Metabolite extraction. The extracted and derivation methods for the metabolites were used as described by Liu et al., with slight modifications [21]. After the cells were treated, the culture medium was removed; 1 mL of chilled (4 °C) water was added, and the cells were washed twice gently. Then, 1 mL of −20 °C prechilled methanol/water (3:2, *v*/*v*) was added to quench the cell activity, and the cells were scraped off gently with a sterile scraper and collected in an Eppendorf tube. This process was repeated twice, and the samples were held on ice. The samples were centrifuged at 13,500× *g* at 4 °C for 2 min, then the precipitated cells were collected. Up to 500 μL of chilled acetonitrile/isopropanol/H_2_O (3:3:2, *v*/*v*/*v*) and two clean stainless-steel beads were added, and then vortexed for 15 s. The cells were homogenized using eight cycles of GenGrinder at 1350× *g* for 30 s, followed by 3 min of sonication. The cells were centrifuged at 13,500× *g* for 2 min at 4 °C, and the supernatant was transferred into a new Eppendorf tube. This extraction procedure was repeated three times. Each group was designed with six replicates, and one quality control (QC) was used for every six samples. A blank sample without cells was used to calibrate the background signals. The protein concentration in each supernatant sample was determined to standardize the data. All the samples were vacuum-freeze-dried completely for further derivatization.

Derivatization. An aliquot of a fatty acid methyl ester mixture (C8–C16: 1 mg/mL; C18–C24: 0.5 mg/mL in chloroform) was used as the internal standard and then added to the samples. Up to 10 μL of O-methoxyamine hydrochloride (dissolved in pyridine) with a final concentration of 0.02 g/mL was mixed with the samples and derivatized for 20 min at 80 °C. Subsequently, 80 μL of MSTFA was added to each tube after cooling. Then, the tubes were sealed promptly and incubated at 37 °C for 90 min. The samples were transferred to a chromatographic injection vial for the GC–TOF/MS analysis.

GC–TOF/MS conditions. A Pegasus BT GC–TOF/MS (LECO, Joseph, MI, USA) coupled with a DB–5 MS column (30 m × 250 μm id, 0.25-μm film thickness; Agilent Technologies, Palo Alto, CA, USA) was used for sample analysis. The details of the GC–TOF/MS detection are described in our previous publication [22]. Solvent delay time was set as 4 min.

### 2.5. Metabolite Profiling Analysis

The raw data were converted twice using ChromaTOF software from LECO as well as the ABF converter to obtain the “mzXML” and “abf” formats. The files in the “abf” format were analyzed using MS DIAL software with the Fiehn library, including filtration and calibration of the baseline, peak alignment, deconvolution analysis, peak identification, and integration of peak height [23]. The parameter settings for peak detection were carried out following a report by Wang et al. [24], which will not be described in detail here. The peak area for each detected peak of each sample was normalized using the SERRF normalization method based on the QC samples, and the normalized data were used for the subsequent multivariate analysis.

### 2.6. Multivariate Analysis

SIMCA 14.1 (Umetrics, Malmo, Sweden) and R i386 3.6.3 software, MetaboAnalyst 4.0 platform were used to carry out multivariate statistical analysis, including a principal component analysis (PCA, including score plot and loading plot) and orthogonal projection to latent structure discriminant analysis (OPLS-DA), a heatmap analysis, and a pathway enrichment analysis. Pathway mapping was analyzed using MetaMapp 2020 platform and generated using CytoScape 3.7.2 (Boston, MA, USA). Significant differences between the control and treated groups were analyzed by one-way ANOVA and *t*-test using SPSS 20 software (SPSS Inc., Chicago, IL, USA). Biomarkers were analyzed using S-plot in the OPLS-DA model. The area under the receiver operating characteristic curve (ROC) curve was computed based on two models using MetaboAnalyst 4.0 and SPSS 20.0 software to evaluate the classification performance.

## 3. Results and Discussion

### 3.1. Optimal Cell Line for Oxidative Stress Damage Model

**Figure 1:**
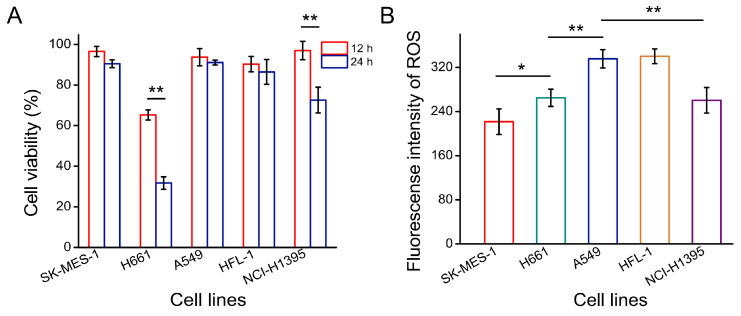
(**A**) Cell viabilities of the five lung cancer cell lines treated with PQ. Results were expressed as percentage compared with each control group under different treatment time, and the cell viabilities of all the control groups were set to 100%. (**B**) Fluorescence intensity values of ROS in different cell lines treated with PQ. The fluorescence intensity of control group was used as a background baseline. * means *p* < 0.05 and ** means *p* < 0.01. All experiments were repeated three times and results were expressed as means ± SD.

Lung tissue has a unique redox environment, as it is easily exposed to various exogenous and endogenous oxidative inducers of oxidation that contribute to the frequency and promotion of inflammation and even tumors [25,26,27,28]. Using this, the cytotoxicity of several lung cell lines treated with 100 μM PQ as an oxidative stress inducer was measured at different times. As shown in Figure 1A, H661 cells were sensitive to the PQ treatment, and they reached their lowest cell survival rate after the 12 h and 24 h treatments (65.25 ± 2.53% and 31.70 ± 3.09%, respectively). NCI-1395 cells were not significantly different from the control group after induction for 12 h, but the survival rate significantly decreased after a prolonged treatment (72.57 ± 6.33% for 24 h). The survival rate of SK-MES-1, A549, and HFL-1 cells remained ≥85% after 24 h treatment, which was suitable for the oxidative stress cell model. Furthermore, the ROS production of the five cell lines induced by PQ showed that A549 cells and SK-MES-1 cells had a higher ROS fluorescence intensity (3.15- and 3.40-fold compared with the control group, respectively) (Figure 1B). Considering the relatively short proliferation cycle, A549 cells were selected to study the antioxidant effects and mechanism of Del.

### 3.2. Multivariate Statistical Analysis to Visualize Metabolites

The metabolic regulation of Del in A549 cells was investigated at 40 μM under the oxidative stress induced by PQ (100 μM). The GC-TOF/MS analysis detected a total of 166 metabolites in the control group, Del pre-protected group, and PQ-induced group after the identification and quantification of the mass spectra using the software and an in-house library. A multivariate analysis method was performed to compare the metabolic perturbations in the three experimental groups. The metabolite profiles of A549 cell samples were analyzed by PCA and OPLS-DA analysis to demonstrate the differentiation in the metabolite profiles between the control group and treated groups.

**Figure 2:**
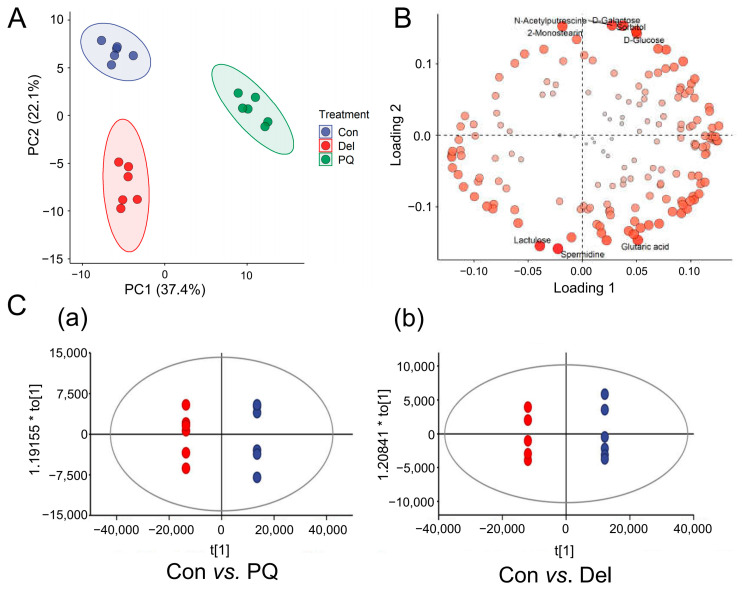
Multivariate statistical analysis of different treatment groups. (**A**) PCA score plot. Variance of PC1 was 28.5%, and variance of PC2 was 19.6%. (**B**) PCA loading plot. (**C**) Score plot from OPLS-DA models. (**a**) PQ vs. control group and (**b**) Del vs. control group.

The PCA results showed that the metabolites in the PQ-induced group were distinctly separate from those in the control group, and the cluster of dots in the Del pretreatment group was closer to the control group (Figure 2A). This observation suggests that the PQ treatment indeed altered the metabolism of A549 cells, and that pre-protection with Del reversed this perturbation to a certain extent. The first two principal components of the treatment groups explained 48.1% of the total variation (63.68% and 52.13% for the PQ group and Del group compared with the control group, respectively). The PCA loading plot further indicated the inconsistencies in the metabolic profiles in these treatment groups based on the relationship between the first two principal components and the metabolite level, consistent with the PCA results (Figure 2B). The control group and PQ group showed a clear intergroup separation along PC1, indicating significant variations in the level of metabolic components, and the model was successfully verified. This finding further indicates that the changes in the metabolic profile, which can be attributed to the induction of PQ and protection by Del, were reproducible.

**Figure 3:**
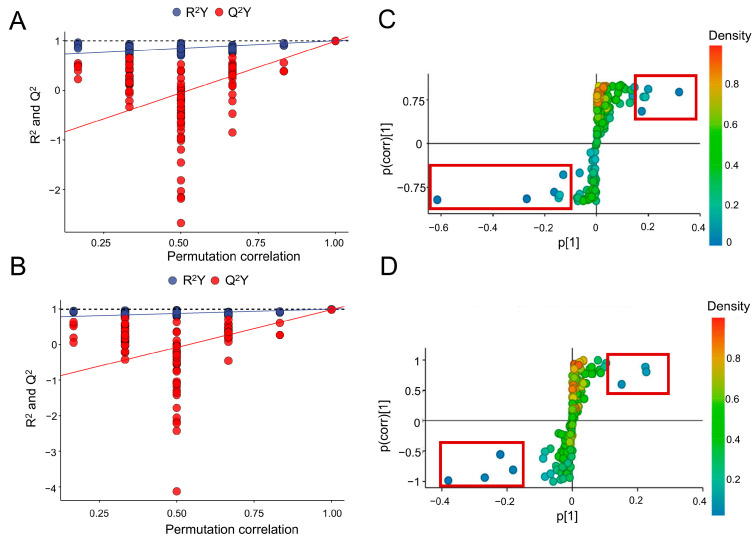
OPLD-DA analysis of metabolites in PQ-induced A549 cells with or without Del. (**A**) and (**B**) The permutation results with 200 iterations of metabolites in PQ and Del treatment groups. The corresponding intercept values of R^2^ were 1 and 1, Q^2^ were −0.429 and −0.263, respectively. (**C**) and (**D**) S-plot of OPLS-DA analysis of metabolites in PQ and Del treatment groups. Metabolites in the left bottom and right top of the S-plot were potential biomarkers.

Two OPLS-DA models were successfully obtained by modeling the first two principal components after PQ and Del groups were compared with the control group (Figure 2C). The interpretation rates (R^2^ X = 0.776 and 0.691, R^2^ Y = 0.994 and 0.997 for the PQ and Del groups, respectively) and the prediction (Q^2^ = 0.981 and 0.973 for the PQ and Del groups, respectively) showed that the OPLS-DA model has good fitness and predictability. Permutation tests with 200 iterations were performed to check the validity and avoid overfitting [29]. As shown in Figure 3A,B, R^2^ (blue) and Q^2^ (red) values to the left were lower than the right points. The R^2^ intercept values were 1, 1, and the Q2 values were −0.429 and −0.263 for the PQ and Del groups, respectively, further indicating a verified model. The metabolites extracted from treated A549 cells showed inconsistencies in the metabolic profiles.

**Figure 4:**
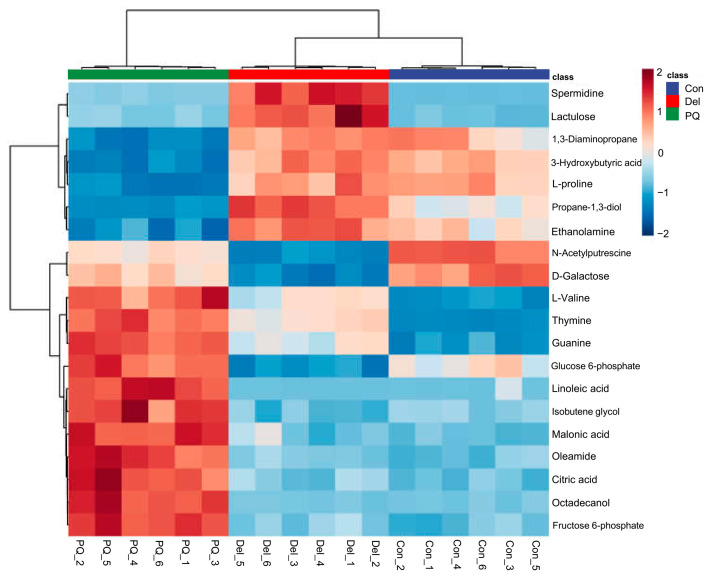
Heat map of the cluster analysis of the first 20 differential metabolites of A549 cell treated with PQ and Del. These differential metabolites were screened with FC > 1.2 and *p* < 0.05.

A heatmap was also constructed to understand the differences and similarities among the control group and the two treated groups (Figure 4). To clearly present the discrepancies in the metabolite concentrations of different groups, the first 20 metabolites were selected for hierarchical Pearson clustering. The hierarchical Pearson’s clustering analysis indicated differences between the control and PQ groups. The color changes in the Del treatment group were closer to those observed in the control group, consistent with the results of PCA and OPLS-DA analyses. The intensity of these metabolites was reversed to a certain extent by the Del pretreatment. Among all significant metabolites in the PQ group, 7 metabolites exhibited a decreasing trend and 11 metabolites exhibited an increasing trend, while these disturbances were recovered to varying degrees after the Del pretreatment.

PQ treatment significantly downregulated the L-proline level in A549 cells. L-proline is a multifunctional amino acid that plays an important role in various important physiological activities, such as primary carbon and nitrogen metabolism, oxidative stress protection, cell signal transduction, and programmed cell death [30]. L-proline has been shown to protect animals, microorganisms, and plants from oxidative stress, possibly by participating in ROS scavenging, increasing antioxidant related enzyme activity, and maintaining the levels of key redox equilibrium molecules such as glutathione and nicotinamide adenine dinucleotide phosphate (NADPH/NADP^+^) [31,32,33,34]. Moreover, PQ also decreased the level of 3-hydroxybutyric acid, a normal metabolite of fatty acid oxidation, which can be used as an energy resource in the case of hypoglycemia [35]. Studies have shown that 3-hydroxybutyric acid can reduce the level of ROS, nitrite, and lipid peroxidation, increase the levels of glutathione, and improve mitochondrial function in hippocampal HT-22 cells [36]. The significant upregulation of 3-hydroxybutyric acid may be related to the antioxidant activity of Del.

Moreover, PQ significantly increased the levels of L-valine, linoleic acid, malonic acid, and citric acid. A study has shown that high L-valine level is associated with increased oxidative stress, thus promoting insulin resistance and newly diagnosed type 2 diabetes mellitus [37]. Linoleic acid is an essential fatty acid, but its high level of dietary and in vitro cellular exposure has been shown to increase oxidative stress [38,39]. Malonic acid can induce Huntington-like behavioral and mitochondrial alterations by significantly causing oxidative stress damage in rat ipsilateral striatum [40]. Furthermore, glucose 6-phosphate and citric acid are important substances involved in energy metabolism, and their abnormal accumulation suggests that energy metabolism pathway may be disturbed. For example, the activities of glucose-6-phosphate dehydrogenase in pentose phosphate pathway and aconitase in citric acid cycle (TCA) affected the metabolism of glucose -6-phosphate and citric acid [41,42]. These speculations require further study.

### 3.3. Identification of Different Metabolites and Prediction of Biomarkers

#### 3.3.1. Differential Metabolite Screening

Furthermore, the variable importance for the projection (VIP) obtained by the S-plot analysis of the OPLS-DA model (Figure 3C,D) was combined with the FC value and *p* value obtained by volcano plot analysis. The metabolites with VIP value > 1.0 and *p* < 0.05 in two treatment groups were screened, and the corresponding FC values were summarized. Twelve and six significant metabolites (FC > 1.5 or FC < 0.65) were obtained in the PQ and DEL treatment groups, respectively, as potential metabolite biomarkers, as shown in Table 1. Five amino acids, three fatty and fatty acids, one carbohydrate, and other metabolites related to proteins and energy metabolism were identified in the PQ group. In the Del pretreatment group, four amino acids, two carbohydrates, and other metabolites were involved in energy metabolism. These different metabolites varied between the PQ-induced group and Del pretreatment group.

The abundance levels of eight metabolites were higher than those of the control group in the Del treatment group, while the abundance levels of three metabolites were downregulated (Table 1). Notably, other significant metabolites were also observed in the Del group, including L-lactic acid, L-Threonine, sulfate, and succinic acid. In addition, D-galactose and D-glucose in the Del group significantly decreased with FC values of 0.36 and 0.53 (*p* > 0.01), respectively. This result indicates that Del was not only involved in regulating the metabolic pathways induced by PQ, but also responded to other metabolic processes. The S-plot of OPLS-DA model showed the clusters of the three groups, which was consistent with the one-way ANOVA result, and further identified the differences between the groups (Figure 3C,D).

#### 3.3.2. Prediction and Verification of Biomarkers

Since a single differential metabolite or combination of metabolites can be used as biomarkers, the area under the ROC (AUC) value (>0.7) of each differential metabolite was calculated by using SPSS20 software to screen single biomarkers. The results show that six metabolites were obtained in the PQ-treatment group, including alanine, valine, urea, thymine, maleimide, and isobutene glycol (AUC = 0.733–0.917). Five metabolites were screened in the Del pre-protected group: alanine, threonine, thymine, galactose, and glucose (AUC value was 0.722–0.889).

**Figure 5:**
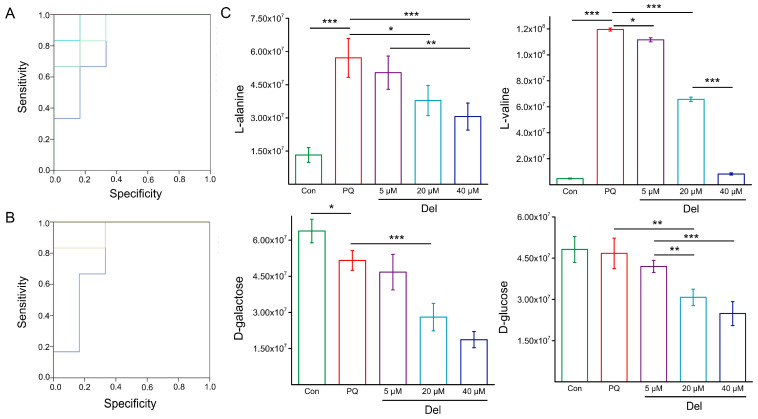
Prediction and validation of biomarker metabolites. (**A**) Predicted ROC curves of biomarker metabolites in PQ treated group. (**B**) Predicted ROC curves of biomarker metabolites in Del pre-protected group. (**C**) Effects of Del pre-protection on the levels of the four metabolic biomarkers. * means *p* < 0.05, ** means *p* < 0.01, and *** means *p* < 0.001.

Further, these metabolites (AUC value > 0.7) were calculated using a binary logistic regression model to obtain the combined factor variable values of different combinations. Then, a predictive ROC curve of biomarkers for multifactor joint evaluation was plotted, and the corresponding AUC values were obtained (as shown in Figure 5A,B). The combinations of metabolites were sorted according to the AUC value, which was used as the optimal combination for evaluating PQ-induced oxidative stress and antioxidant activity of Del (Table 2 shows the three combinations with the largest AUC values in the two treatment groups). As shown in Table 2, the combination of three metabolites was optimal in each treatment group. The selected combination of biomarkers in PQ-treatment group was alanine-valine-urea (AUC = 0.972), which was mainly involved in the metabolism of amino acids. A significant effect on glycometabolism and amino acid metabolism was found in the Del pre-protected group, and the combination of alanine-galactose-glucose afforded the optimal evaluation and prediction effect (AUC = 0.998) (all confidence interval was <0.75).

The AUC values of alanine with a high predictive accuracy were found in both the treatment groups (Table 2). Neutral amino acids such as alanine, glycine, and serine can protect the liver and kidney cells from various oxidative, metabolic, and chemogenic damage [43]. Alanine and its similar amino acids can effectively reduce cell apoptosis caused by the increase of the ROS or calcium ion levels. Meanwhile, alanine protects cells by regulating the signaling pathways and genes related to the intracellular oxidative defense system, such as heme oxygenase-1 and ferritin (a key catalyst for the formation of oxygen-centered free radicals through Fenton reaction) [44]. When cells are subjected to oxidative stress, the intracellular alanine level is significantly increased. In addition, alanine is involved in the intracellular glycolytic energy supply pathway, and the accumulation of alanine may lead to an abnormal energy metabolism pathway. Combined with the FC value of alanine and the antioxidant activity of Del shown in Table 1, alanine could be used as a cobiomarker for the evaluation of antioxidant activity in a PQ-induced oxidative stress cell model.

To verify the accuracy of biomarker combination, the level of biomarkers under 5, 20 μM of Del was studied, and the results are shown in Figure 5C. Compared with the control group, the levels of L-alanine and L-valine were significantly upregulated in the PQ-induced group, while the levels of D-glucose and D-galactose were moderately downregulated. In the Del pre-protected group, with the increase in the concentration of Del, the change trend of L-alanine and L-valine was closer to that of the control group, but the levels of D-glucose and D-galactose were continued to decrease significantly in a dose dependent manner. These results show that the identified biomarkers had excellent predictive accuracy.

### 3.4. Metabolic Pathway Analysis

**Figure 6:**
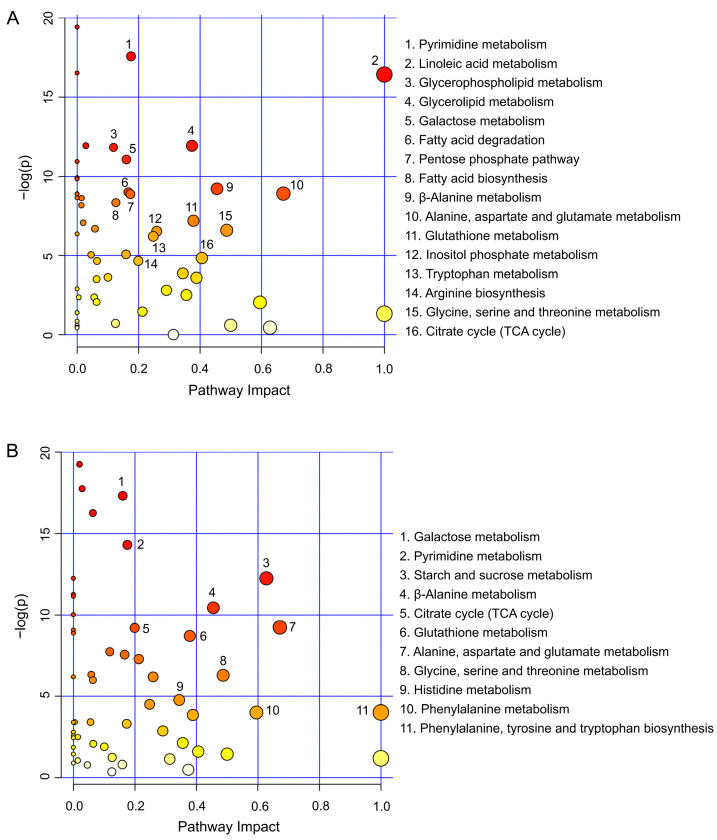
Enrichment of metabolic pathways in A549 cells treated with (**A**) PQ and (**B**) Del. The larger circle and darker color represented the greater impact on metabolic pathways. The X axis represents the impact factor of the pathway topology analysis and the Y axis represents the *p* value of the pathway enrichment analysis.

To analyze the effects of intracellular oxidative stress environment on metabolic pathways induced by PQ with or without Del pretreatment, metabolites with FC values < 0.65 and >1.5 in the two treatment groups were screened, and Venn analysis was performed to remove the repeated values. The set of metabolites was mapped using MetaboAnalyst 4.0 to determine whether the screened metabolites would map to pathways related to the biochemical and physiological changes that occurred in the groups after the PQ and Del treatments. Several metabolic pathways were identified when A549 cells were treated with PQ and significantly enriched from the oxidative stress perspective. These pathways were involved in linoleic acid, pyrimidine, glycerolipid, glutamate, galactose, glutathione, and metabolism of several amino acids, TCA cycle, arginine, phenylalanine, tyrosine, and tryptophan biosynthesis, which participated in the metabolism of energy, amino acids, and lipids (Figure 6A). The enrichment results of metabolic pathways were consistent with the screening results of differential metabolites described above.

Many studies have shown that stress response or disease can affect amino acid metabolism, energy metabolism, and lipid metabolism [45,46,47]. Amino acids such as glutamate, aspartate, phenylalanine, tyrosine, and leucine are pivotal metabolic intermediates in the central carbon metabolic pathway, including TCA cycle and pentose phosphate pathway [48]. For example, pyruvate, a-ketoglutarate, and oxaloacetate, which are important substances involved in the pentose phosphate pathway, were formed by transamination of alanine, glutamate, and aspartate, respectively [49]. Therefore, the imbalance of amino acid metabolism will directly or indirectly affect the energy metabolic pathway, while the disruption of energy homeostasis is a potential marker of inflammation, oxidative stress damage, and even disease [50]. The arginine biosynthesis pathway was enriched in PQ treatment group. This may be attributed to the significantly increased level of L-valine which inhibits arginase activity, thus, reduces the arginine level. L-valine is a known inhibitor of arginase [51]. Moreover, intracellular β-oxidation has been reported to generate ROS. Mitochondrial β-oxidation is responsible for the degradation of short, medium, and long-chain fatty acids, while peroxisomal β-oxidation of long, very long, and branched-chain fatty acids, which are associated with the increase level of intracellular ROS [52]. Fatty acids can be broken down into acetyl-CoA and enter the TCA cycle to participate in the ATP production [53]. Meanwhile, fatty acids are also involved in the formation of more complex lipids. In the PQ treatment group, several pathways related to lipid metabolisms were enriched, such as linoleic acid, glycerolipid, and glycerophospholipid metabolisms, and fatty acid degradation and biosynthesis. These results suggest that PQ induction significantly disrupt metabolism in A549 cells.

However, the A549 cells pretreated with Del reversed the significant metabolic effects induced by PQ, with several metabolic processes returning to levels similar to those of the control group (Figure 6B). Pathways such as beta-alanine metabolism, phenylalanine, tyrosine and tryptophan biosynthesis, TCA cycle, starch and sucrose metabolism, pyrimidine metabolism, alanine, aspartate, and glutamate metabolism, and glutathione metabolism were enriched. These results also suggest that Del protected A549 cells from oxidative stress by regulating multiple metabolic pathways, consistent with the aforementioned cellular antioxidant results.

**Figure 7:**
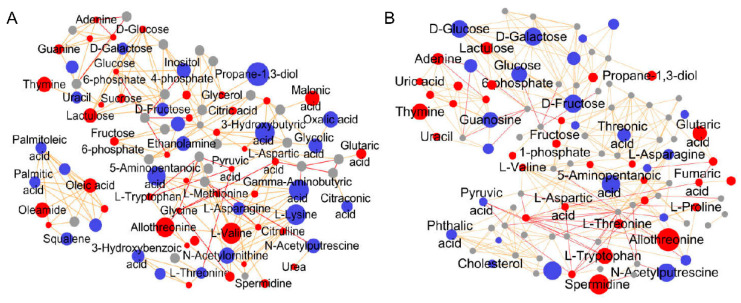
Metabolic pathway network map of A549 cells treated with (**A**) PQ and (**B**) Del. Blue, red, and gray colors represented the downregulated, upregulated and unaffected metabolites, respectively.

To further evaluate possible metabolite–metabolite interactions, a metabolic pathway map, potentially influenced by PQ induction and Del pre-protection, was analyzed using the MetaMapp platform and generated using CytoScape 3.7.2 (Figure 7). The diameter of graph is related to the FC value and the *p*-value of the *t*-test. The PQ treatment affected several metabolic processes in A549 cells. The level of metabolites such as citrulline, L-alanine, L-serine, L-valine, propane-1,3-diol, malonic acid, 2-monoolein, and oleic acid increased significantly (*p* < 0.001) compared to those in the control group, whereas squalene, 1-monopalmitin, glycolic acid, D-galactose, and 3-hydroxybutyric acid (*p* < 0.01) significantly decreased (Figure 7A). Treatment with Del reversed the PQ-induced changes (Figure 7B) similar to the control group, indicating that Del reversed the metabolic disturbance caused by PQ and significantly maintained the normal metabolic dynamics in A549 cells. Interestingly, the abundance levels of D-glucose and D-galactose significantly decreased in the Del treatment group and combined with the enriched pathway of starch and sucrose metabolism, indicating that Del also resisted oxidative stress by affecting glycometabolism. These analytical results support the previous results, indicating that the metabolic pathways in A549 cells changed after the PQ treatment and were effectively reversed by Del.

Amino acids play important roles in purine and sphingolipid biosynthesis, cell metabolism, and neurotransmitter synthesis associated with oxidative stress and aging [54,55,56]. For instance, sulfur-containing amino acids play key roles in the metabolic adaptations to oxidative stress [57,58]. The PQ and Del treatments did not completely interrupt the energy supply process but rather replenished it via the glycolytic pathway to resist oxidative stress. The disturbance in the TCA cycle and pentose phosphate pathway induced by PQ led to a reduction in mitochondrial ATP synthesis, affecting energy generation [59,60,61]. However, the PQ treatment had no significant effect on glycometabolism, whereas the Del pretreatment upregulated or downregulated the abundance of several metabolites such as fructose, lactulose, and glucose. Moreover, the abundance levels of octadecanol, glycerophosphoric acid, glycerol, and 2-monoolein significantly increased in the PQ group, whereas those of squalene, 1-monoolein, and glyceric acid significantly decreased relative to those in the control group (*p* < 0.05) (Table 1). These results indicate that the lipid metabolism was affected by oxidative stress which integrated with the enriched pathways. Del reversed the changes in fatty acids and lipids induced by PQ, indicating that Del may reduce the incidence of obesity [62,63]. Unsaturated fatty acids are intracellular scavengers, which illustrates the relationship between the reversal in the abundance of the unsaturated fatty acids and the antioxidant activity of Del [64].

A549 cells pretreated with Del generally changed the cellular metabolic perturbation effect of PQ, particularly the metabolites associated with oxidative stress. The intracellular antioxidant effect of Del is a complex process, which requires the mobilization of multiple pathways. The metabolic pathway analysis and differential metabolite results showed that the Del pretreatment caused the least metabolic disturbance and the PQ treatment had the greatest effect compared to the control group, consistent with the previous antioxidant activity results. The metabolites reflected the results of perturbations in A549 cells induced by various stressors, which may be overlooked by traditional antioxidant evaluation methods.

## 4. Conclusions

In this study, A549 cell line was selected to establish a PQ-induced oxidative stress cell model; then, the potential mechanism of Del on the oxidative stress of A549 cells was preliminarily investigated using cell metabolomics analysis combined with GC-TOF/MS. The Del pretreatment alleviated the disorders of tricarboxylic acid cycle, energy metabolism, amino acid metabolism, lipid metabolism, and glucose metabolism which induced by PQ. Alanine-galactose-glucose was identified as a biomarker combination of antioxidant activity of Del. The results indicate that that Del can maintain the metabolic characteristics of A549 cells related to normal redox homeostasis, and it probably provides a characteristic fingerprint associated with cellular antioxidant activity. This analysis of the potential antioxidant mechanism of Del based-on metabolomics will help in understanding the Del response to relevant metabolic pathways after oxidant exposure and provide potential screening biomarkers for the antioxidant activity evaluation of Del.

## Figures and Tables

**Table 1:** Statistical analysis of differential metabolites in PQ and Del treatment groups.

Number	Metabolites	PubChem ID/ *	KEGG ID	Fold Change Value	
				PQ	Del
1	Propane-1,3-diol	10,442	C02457	3.93 **Δ	1.87 **Δ
2	L-Alanine	5950	C00041	3.93 **Δ	2.49 **Δ
3	Oxalic acid	971	C00209	0.59 **Δ	0.94
4	D-Galactose	6036	C00984	0.79 **Δ	0.36 **Δ
5	Maleimide	10,935	C07272	2.87 **Δ	1.10
6	L-Valine	6287	C00183	25.95 **Δ	1.20 Δ
7	Urea	1176	C00086	1.54 *Δ	1.04
8	Malonic acid	867	C00383	5.13 **Δ	1.21
9	Isobutene Glycol	68,410	C21290	0.65 **Δ	1.07
10	Thymine	5610	C00483	5.30 **Δ	3.65 **Δ
11	Oleic acid	445,639	C00712	3.91 **Δ	1.21
12	3-Hydroxybutyric acid	441	C01089	0.26 **Δ	1.14
13	L-Threonine	6288	C00188	0.40 **Δ	1.83 **Δ
14	D-Glucose	5793	C00031	1.06	0.53 **Δ

Δ means VIP > 1 in OPLS-DA. * means *p* < 0.05 compared with control group; ** means *p* < 0.01. compared with control group. KEGG is short for Kyoto Encyclopedia of Genes and Genomes.

**Table 2:** List of the screened biomarker metabolites.

Combination	PQ Group vs. Con Group		Del Group vs. Con Group	
	Metabolites	AUC Value	Metabolites	AUC Value
1	L-alanine/L-valine	0.944	L-alanine/L-Threonine	0.889
2	L-valine/urea	0.972	D-galactose/D-glucose	0.944
3	L-alanine/L-valine/urea	0.972	L-alanine/D-galactose/D-glucose	0.998

AUC in the table means the area under the receiver operating characteristic curve.

## Data Availability

Data are contained within the article.

## Abbreviations

AUC	area under the ROC
Del	delphinidin
FC	fold change
GC-TOF/MS	gas chromatography-time of flight mass spectrometry
KEGG	Kyoto Encyclopedia of Genes and Genomes
NADPH	nicotinamide adenine dinucleotide phosphate
OPLS-DA	orthogonal projection to latent structure discriminant analysis
PCA	principal component analysis
PQ	paraquat
QC	quality control
ROC	receiver operating characteristic curve
ROS	reactive oxygen species
TCA	citric acid cycle
VIP	variable importance for the projection
